# The Pheromone Landscape of *Apis mellifera*: Caste-Determined Chemical Signals and Their Influence on Social Dynamics

**DOI:** 10.3390/molecules30112369

**Published:** 2025-05-29

**Authors:** Anna Gryboś, Patrycja Staniszewska, Maciej Sylwester Bryś, Aneta Strachecka

**Affiliations:** Department of Invertebrate Ecophysiology and Experimental Biology, University of Life Sciences in Lublin, Doświadczalna 50a, 20-280 Lublin, Poland; grybosanna156@gmail.com (A.G.); maciej.brys@up.lublin.pl (M.S.B.); aneta.strachecka@up.lublin.pl (A.S.)

**Keywords:** honeybee, QMP, sensilla, pheromone glands, chemical communication

## Abstract

A honeybee (*Apis mellifera*) colony is a superorganism of complex social dynamics. Within the colony, communication between individuals and castes is crucial for maintaining homeostasis. Such complex interactions are possible thanks to semiochemicals called pheromones. The spectrum of pheromonal communication in bee colonies is wide and differs between castes, especially the queen and the workers. Gland morphology and compounds of secretions result in alterations in both physiological and behavioral responses to certain pheromones in castes. The queen’s glands produce pheromones that maintain her reign and induce division of labor among workers. Workers’ pheromones are adapted to multiple tasks performed by this caste within the colony. This review outlines a neurophysiological pathway in the perception pheromone molecule, with a specific description of the individual anatomical structures essential for the path, such as the morphology of antennae, sensilla, antennal lobes and mushroom bodies. Later on, the study provides insight into specific aspects of the differences between the two castes (queen and workers) in terms of complex pheromonal communication in the hive, by describing the pheromones present in it (QMP, tergal gland pheromone, Dufour gland pheromone, Nasonov pheromone, sting alarm pheromone and tarsal gland pheromone).

## 1. Introduction

The honeybee (*Apis mellifera*) is a species that has been a companion to humans since the dawn of time. The fruits of its labor are used by humans in almost every aspect of life, from food products to cosmetology to medicine. However, the bee is an important invertebrate not only because of its association with industry but primarily because of its indispensability to the environment. The honeybee’s anatomy, physiology and behavior are constantly being discovered, and despite the small size of this insect, the degree of its complexity is surprising. The honeybee is an insect classified as a eusocial, that is, an insect that lives in colonies with a hierarchical division. This includes workers (the most numerous), the queen and drones (males). Each caste performs specific roles in the colony. Among workers, the division of responsibilities is also related to their age (age polyethism) [1,2]. The interactions between individuals/castes, division of duties and other important aspects of work are constantly regulated by compounds called pheromones. They are classified as “semiochemicals”. A pheromone is defined as a mixture of compounds that has the ability to produce a specific physiological and behavioral effect in another individual of the same species [3,4,5]. This publication outlines the crucial value of pheromones to the functioning of a colony as a superorganism. The scientific literature outlining the effects of pheromones on the honeybee leaves many questions unanswered. Much of the baseline information comes from experiments conducted many years back (relative to current scientific developments). Therefore, our article summarizes existing knowledge on the subject and points out gaps that need to be filled.

## 2. Neurophysiology of the Pheromone Pathway

**Figure 1:**
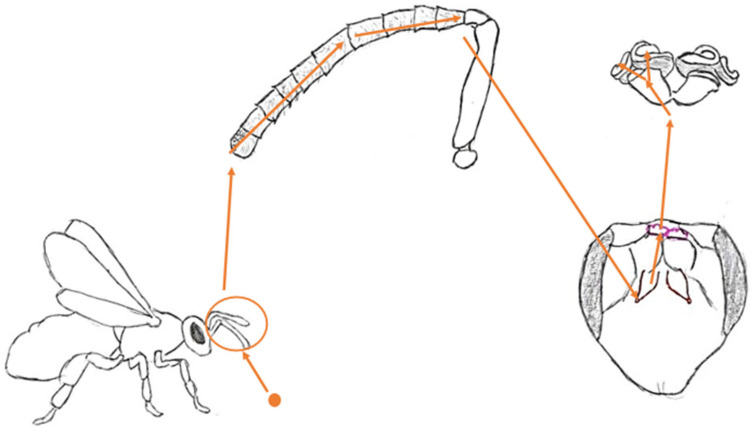
A pictorial pathway of a worker bee’s perception of an odor molecule.

Pheromones, as odor molecules, are perceived and processed thanks to specific anatomical structures. They are located around the insect’s cephalic segment. The molecules themselves do not travel beyond the sensilla; instead, the chemical signal is converted into neuronal impulses that pass through neurons to the antennal lobe and mushroom bodies (Figure 1).

### 2.1. Morphology of Antennae

In honeybees, the sensilla are a key sensory organ for receiving a variety of stimuli in almost every aspect of life. Both of the following are associated with the stimuli received by sensory organs: responding to pheromones, but also to other variable environmental factors affecting behavior. This role enables the survival of an individual as well as an entire colony. The sensilla in insects are well-studied organs for receiving gustatory and olfactory stimuli. However, their reactions are not limited to responses to these stimuli. Signals received by these organs include changes in air temperature (thermosensors) and wind strength [6,7].

**Figure 2:**
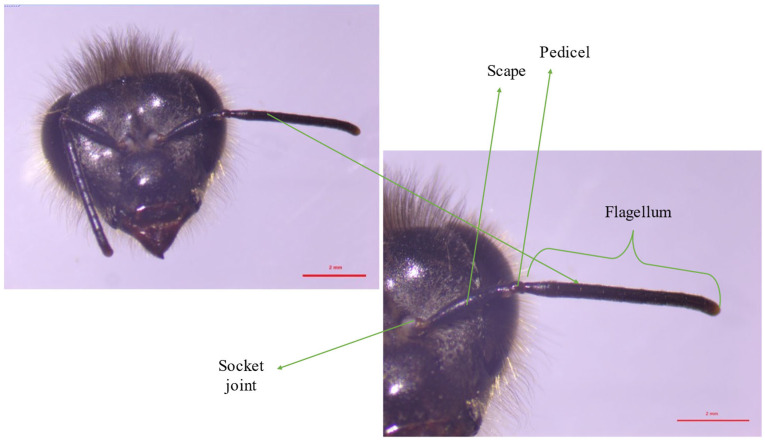
Structure and location of the worker bee’s antennae.

Antennae are divided into four components (socket joint, scape, pedicel and flagellum) (Figure 2). Each is covered with hairs [8]. The antennae are mobile organs, thanks to muscles (in sequence: flexor, extensor, depressor and jack) attached to the scape by tendons. They have the ability to move vertically, as well as partially laterally. Four muscles out of six are located in the socket joint and are responsible for moving the scape [9]. The scape is rigid and straight, but the movements made by the antennae are made possible by embedding the scape spherically in the socket joint. Inside it are two antagonistic muscles. Their contractions allow upward and downward movement of another two structures—the pedicel and flagellum. The flagellum consists of many annuli. Their number differs between drones (11/12 annuli) and workers (10/11 annuli) [10,11,12]. The movements of these organs allow the sensilla to contact the odor molecule.

### 2.2. Sensilla

Sensilla are located on the surface of the above-described flagellum, actively receiving stimuli of smell, taste, changes in wind strength, humidity and ambient temperature. Due to their structure as well as the type of stimulus they receive, they have been divided into eight types. These include (1) trichodea (divided into three sub-groups, A, B, C and D or I, II, III and IV), (2) placodea, (3) coeloconica, (4) coelocapitular, (5) campaniform, (6) basiconica, (7) ampullacea and (8) chaetica. Each of the types has a specific location on the flagellum. Sensilla trichoidea are found on every annulus of the flagellum; however, their sub-group density varies. Only the dorsal ends of the first through third segments and the first through second segments on the ventral side include sensilla trichodea type A. On the dorsal side of the second to tenth segments and the ventral side fourth to tenth segments of the flagellum, sensilla trichodea type B is found. Particularly toward the final segments, this type is more prevalent on the flagellum’s distal ends than its proximal ends. On the dorsal side of the third to tenth segments and on the ventral side of the fourth to tenth segments, sensilla trichodea type C is found. On the third to tenth segments of the dorsal and ventral sides, sensilla trichodea type D is visible. Additionally, there are also a lot of setae on the flagellum’s ventral side. On the dorsal side of the ninth through tenth segments and on the final segment on the ventral side, sensilla campaniforme can be seen sporadically. Sensilla placodea are found on the third to tenth segments, while ampullacea and coeloconica on flagellomeres six to ten. Sensilla ampullacea do not occur on the ventral side of flagellum. Sensilla basiconica can be found on dorsal side of the fourth and tenth flagellomeres. However, on the ventral side, this type of sensilla can be spotted on flagellomeres eight, nine and ten. Their density increases peripherally [13,14,15] (Table 1).

### 2.3. Odor Detecting Sensilla

In honeybees, three types of sensilla have been identified as olfactory sensilla. Sensilla basiconica (peg-like sensilla), sensilla trichodea (hair-like sensilla) and sensilla placodea (pore plate sensilla) [16]

Sensilla basiconica represent a sex-specific difference between females and drones of *Apis mellifera*. This specific type of sensilla is lacking on the surface of drone’s antennae. Sensilla basiconica are called “peg-like” due to their structure. They occur in the shape of a peg with a nearly flattened end. On the peg surface, sensilla basiconica possess numerous pores. Dendrites of neuron receptors branch within the pores. Outside of its olfactory perception, sensillum basiconca are known as gustatory receptors. Three to five receptor cells innervate each sensillum. Receptor cells are immersed in sensillum lymph [17].

Sensilla trichodea have a hair-like structure and can be divided into four categories (A, B, C and D or I, II, III and IV, depending on the source). Part of the sensillum above the cuticle is called “seta”. With regard to its thickness and degree of bending, a classification was issued. Seta is anchored in a flexible socket, allowing movement and sensory input. Beneath the socket lies the tubular body, connected to dendritic components. Outer dendrites and inner dendrites originate from bipolar sensory cells. The receptor lymph space surrounds the scolopale sheath that surrounds dendrites. Underneath the cuticle, sensory dendrites of the sensory nerve cell amplify the stimulus when the seta is moved. Type A (I) occurs as thin and straight hair-like structures. Type B (II) is thin at the base but narrows at the distal end. The bending of this part is a taxonomic feature. Type C (III) trichodea appear on flagellum as strongly curved and arched. This type of sensilla is considered thin in comparison to Type D (IV) trichodea, which are curved and the thickest ones. Type A (I) trichodea are considered olfactory. The length and thickness of sensilla trichodea varies among individuals [18].

Sensilla placodea play a crucial role in chemical communication and are most abundant on the surface of the flagellum. They are also called pore sensilla because of their structure. A single sensillum is built with a 9 × 6 µm thin oval cuticular plate. Sensilla placodea are shaped in oval plates embedded in the pores of the cuticle. They are classified as odoriferous sensilla and show high sensitivity even to low concentrations of chemical compounds [10,11,19]. Placodea sensilla contain sensillum lymph, olfactory receptors—ORs (plate) and olfactory receptor neurons—ORNs. Their dendrites undergo extensive branching. Each oval sensilla is innervated by 5-35 ORNs, and each one corresponds to an OR [20,21].

### 2.4. Detection of Pheromone by the Sensilla

Through diffusion in the sensillum lymph, odor molecules reach the ORN dendrites. This diffusion is possible thanks to the presence of Placodea pores. Transport of molecules after diffusion toward the ORN occurs after binding odorant-binding proteins (OBPs). In the case of pheromones, a distinct class of OBPs—pheromone-binding proteins (PBPs)—participates in the transport [20,22]. After contact with the dendritic membrane, the molecules interact with specific chemosensory receptors divided into two molecular families: olfactory and ionotropic receptors. Olfactory receptors are seven-membrane proteins with a ligand-binding domain. Despite the infinite possibilities of forming olfactory combinations between an odorant molecule and a ligand, it should be remembered that the degree of affinity is pair-dependent. Therefore, some pairs will be formed even at low concentrations, but it will be the olfactory system in the bee’s brain that determines whether a behavioral or physiological response is triggered [23].

### 2.5. Antennal Lobe

**Figure 3:**
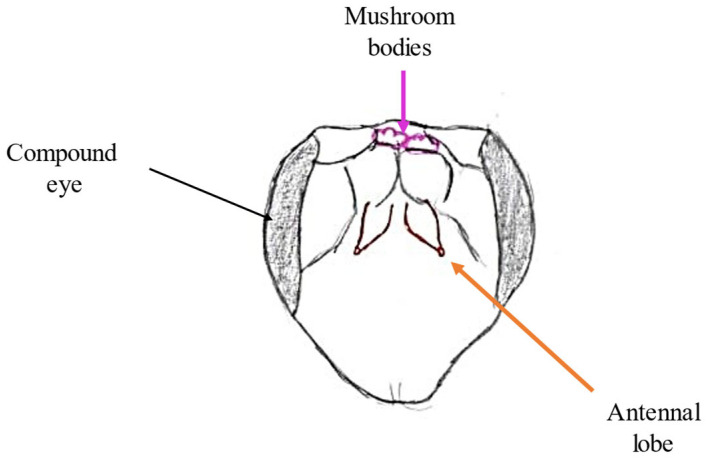
Drawing of the location of the olfactory lobe and mushroom bodies in the bee’s head.

The antennal lobe is part of the deutocerebrum in the bee’s brain responsible for collecting information transmitted by olfactory receptors. The deutocerebrum is one of three neuromeres, i.e., ganglia fused together. The antennal lobe (located in the anterior part) (Figure 3) is a structure containing specific structures—glomeruli (approximately 160). Information collected by olfactory receptors is received and processed by them. Glomeruli are interconnected by a network of neurons. The number of these structures is equal to the number of ORNs that correspond to the OR [10,24]. Odor-responsive neurons characteristically respond to the type of molecule (so-called Odor Identity) and to its concentration. The glomeruli also contain neurons called projection neurons, which transmit the collected information to downstream higher-order units, i.e., the mushroom bodies [25].

### 2.6. Mushroom Bodies and Higher Structures

**Figure 4:**
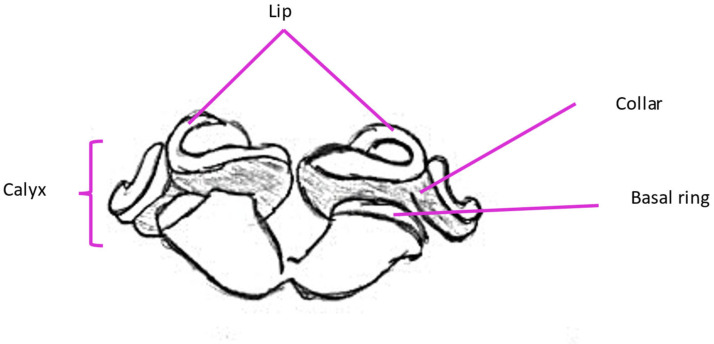
Parts of mushroom bodies responsible for processing olfactory information.

Mushroom bodies are lobed structures located in the brain of the honeybee. They are present as a pair. Their specific regions include Kenyon cells, which form calyces in each cerebral hemisphere. These structures are divided into the lip and the basal ring (olfactory stimulus reception) and the collar (visual stimulus reception) (Figure 4). Kenyon cells transmit olfactory signals via axonal bundles projecting into the midbrain [20,26]. Kenyon cells can be divided into three classes: large-type class I (lKCs), small-type class I (sKCs) and class II. Each of the mentioned types’ somatas are located on different parts of calyces. While both class I somatas are concentrated in the inner part of calyces (lKCs at the inner edges and sKCs in the inner core), class II are located on the outer surface of the mushroom bodies’ calyces. The class division of Kenyon cells is related not only to their size and location but also to their function in mushroom bodies. lKC dendrites extend to particular calyceal sensory subregions, like the visual (collar) or olfactory (lip) areas. Consequently, from the standpoint of pheromone perception, this type is the most significant. sKCs project their dendrites to the basal ring. While the collar and lip are known to be recipients of singular projections, the basal ring has been shown to receive multimodal input in mushroom body output neurons. Dendrites from class II of Kenyon cells extend throughout the calyx [27,28].

Scientific studies have indicated that pheromones may induce behavioral changes by altering gene expression in the brain. Apart from processing olfactory stimuli, mushroom bodies are correlated with learning ability in honeybees and other hymenopterans. lKCs also play a significant part in the learning process of bees. Five genes encode proteins involved in intracellular Ca^2+^-signaling pathways (CaMKII). When CaMKII (calcium- and calmodulin-dependent protein kinase) is activated, it can autophosphorylate. This enables the transition of CaMKII into a constitutively active, calcium-independent state. It is capable of molecular-level information storage thanks to this mechanism. This specific pathway plays a crucial role in learning and memory abilities. It has been established that CaMKII contributes to the development of long-term memory [27,29].

According to research conducted by Privitt et al. [30], the pesticide fipronil (a GABA receptor antagonist) negatively affects the synaptic organization of mushroom bodies and ultimately results in a reduction in the bee population [30].

## 3. Pheromones in the Life of the Honeybee

Pheromones constitute the primary form of communication among individual members of a honeybee colony. Upon reception and processing in the aforementioned olfactory centers, they trigger specific physiological and behavioral responses. In the colony, pheromones are secreted mainly by both the queen and the worker bees. It is worth mentioning that drones also produce pheromones; however, specific information about them is highly limited. Therefore, this paper focuses on the pheromones of the queen and workers. These include the Queen Mandibular Pheromone (QMP), tergal gland pheromone, Dufour gland pheromone, Nasonov pheromone, sting alarm pheromone (SAP) and tarsal gland pheromone. Among these, the QMP is the pheromone that influences the entire colony.

### 3.1. QMP

The Queen Mandibular Pheromone (QMP) is the most well-characterized honeybee pheromone. Its occurrence in the colony was first noticed and described by Colin Gasking Butler. Butler was the first one to use term “queen substance” in 1954 article. The QMP affects the entire colony, influencing every aspect of its functioning. Its compounds induce retinue behavior, swarm clustering, drone attraction, queen rearing, division of labor among workers and their ovary development inhibition. It induces responses through specific neuronal pathways (primer effects) as well as through olfactory perception (releaser effects) [31].

#### 3.1.1. Site of Synthesis

**Figure 5:**
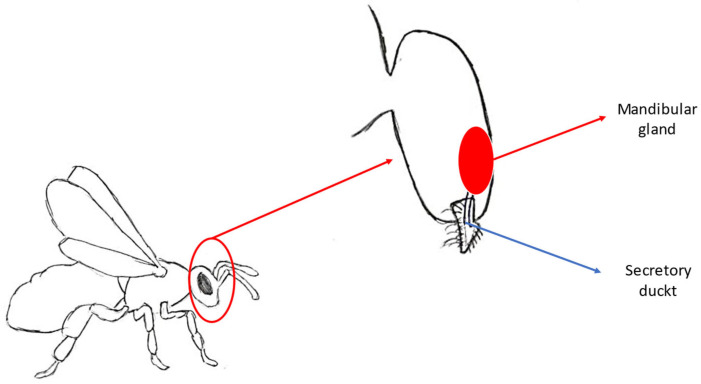
Location of the mandibular gland and secretory duct.

The Queen Mandibular Pheromone is produced in the mandibular glands, which are paired structures located above the base of the mandibles within the head. Mandibular glands belong to class III glands. This type of gland is built with secretory cells and duct cells. Secretory cells contain notable nuclei and cytoplasm plentiful of smooth endoplasmic reticulum. The nuclei of mandibular gland cells are spherical and contains decondensed chromatin. The structure of these cells indicates that compounds synthesized in these glands are lipid-based. When comparing the mandibular glands of queens and workers, a difference in size can be noticed. The secretory cells of the queen’s mandibular glands are larger than the same type of cells in forager workers [32]. The differences in mandibular gland development between castes are a result of differences in feeding larvae with royal jelly. Queens receive larger quantities of royal jelly, of better quality, and are fed throughout their entire lives compared to worker bees. Royal jelly is a secretion that contains proteins, carbohydrates and fatty acids, most prominently 10-HDA (10-hydroxy-2 (E)-decenoic acid). It is fed to all castes of bees in the early larval stage. After the third day of larval development, only future queens stay on a royal jelly-based diet. One of the fatty acid components—10-HDA—has been established to possess a histone deacetylase inhibitor activity (HDACi). This specific activity allows histones to remain acetylated and create a chromatin structure open enough to facilitate transcription of queen-specific genes. A significant chromatic alteration that was shown to have caste-specific areas in honeybees was the implication of histone 3 acetylation at lysine 27 (H3K27) [33,34]. A short duct at the base of the queen’s mandible serves as the gland’s opening, allowing its secretion to flow into another duct surrounded by specialized hairs (Figure 5) [35,36].

#### 3.1.2. Chemical Composition

The Queen Mandibular Pheromone contains several chemical compounds discovered over the years such as: methyl p-hydroxybenzoate (HOB), 9-oxo-2 (E)-decenoic acid (9-ODA), 4-hydroxy-3-methoxyphenylethanol (HVA), (R,E)-9-hydroxy-2-decenoic acid (9-HDA), (S,E)-9-hydroxy-2-decenoic acid (9-HDA), 10-hydroxy-2 (E)-decenoic acid (10-HDA) and 10-hydroxydecanoic acid (10-HDAA) [37]. The first identified component of the pheromone was 9-ODA (9-oxodec-2-enoic acid) and the next 9-HDA (9-hydroxydec-2-enoic acid). However, because these compounds did not correspond to specific glandular activity, they were not considered the only components. A more detailed examination of the chemical composition revealed the presence of three additional compounds in the mixture, including one enantiomer of 9-HDA acid, HOB (methyl p-hydroxybenzoate) and HVA (4-hydroxy-3-methoxyphenylethanol). The percentage of these components in the final product is different for mated and virgin queens. The glands of uninseminated queens, compared to inseminated queens, produce smaller amounts of 9-HDA and 9-ODA (for inseminated queens, it constitutes 80% of the secreted “mother substance”), as well as undetectable amounts of HVA. This means that workers do not perceive them as attractive [38,39]. In comparison, the mandibular glands of workers produce substances that contain several compounds of the QMP, such as 10-HDA and its precursor 10-HDAA, HOB, 9-ODA and 9-HDA. The differences between castes are related to early stages of biosynthesis. Stearic acid, which is the main staring material for the process, goes through hydroxylation in caste-specific locations in the chain (ω or ω-1). During the second stage of the process (β-oxidation), chain shortening of the 18- and 17-hydroxystearic acids occur. The third and last stage is the oxidation of the ω and ω-1 hydroxy group, which results in the production of diacids and keto-acids [37]. The effects of the QMP in a bee colony can be divided into primer (in which it affects specific neuronal pathways) and release (where recognition of the scent induces behavior) effects. Primer effects include building combs, defending the nest, taking care of the offspring by workers, inhibiting the development of worker ovaries and preventing the rearing of new queens. This last effect may be limited in larger colonies, where the pheromone reaches further parts of the colony in a much more diluted form [40].

#### 3.1.3. Juvenile Hormone and QMP

Behind the mushroom bodies in the bee brain are the cell bodies of the secretory organs. One of these organs is the “corpora allata”, which secretes the Juvenile Hormone (JH). One of the theories on how the QMP affects the reduction in the amount of secreted JH is based on its influence on the increase in the concentration of vitellogenin (Vg) in the body. This is possible by stimulating the expression of RNA Vg in the fat bodies of the bees. Vitellogenin inhibits the secretion of JH through a feedback loop. Apart from regulating metamorphosis, JH also influences certain behaviors in bees, such as foraging [41]. McQuillan et al. [42] showed in their studies that JH also affects the expression of amine receptor genes in both antennae and mushroom bodies. Specifically, the impact of higher levels of JH on octopamine receptor gene Amoa1 is connected to pheromones perception. JH treatments have led to decreases in Amoa1 expression in antennae and reduced attraction to the QMP. This allowed us to draw the conclusion that QMP-induced changes in JH titers are likely to contribute to the effects of this pheromone upon the attraction of young bees to the QMP. Therefore, the young bees that gather around the queen are responsible for cleaning and feeding her [42].

#### 3.1.4. Inhibition of Ovarian Development

One of the main effects of QMP in a bee colony is the inhibition of ovarian development in worker bees. As a result, the queen has 150 to 180 ovarioles per ovary, while the worker has approximately 3 to 12 [43]. Since the queen is the only one who lays eggs (in a typical colony without occurring aberrations), inhibiting this ability in other members of the colony allows them to develop altruistic behaviors. The presented action of the queen’s mandible pheromone is more closely related to the initial stage of ontogeny, i.e., the larval stage. Larvae occurring in the hive are fed in two ways depending on their future role. Larvae of future queens are fed with royal jelly, while workers are fed with worker jelly (which contains pollen). On the third/fourth day of life, the larvae are redirected on the developmental path towards the worker or queen, which is influenced by the QMP. The change in the development pathway is related to the transfer of information by workers about the presence or absence of the queen in the nest. This is possible due to trophallaxis (transfer of chemical information resulting from contact between mouthparts and antennae during feeding) [44,45].

The substance that inhibits ovarian development is probably 9-ODA. However, in the course of research, it was theorized that this compound has a smaller effect on inhibiting ovarian development in uninseminated queens than in mated queens. The volatile compound E-β-ocimene is produced by queens after mating, and it also inhibits ovarian development in workers. In addition, the compounds ester palmitate, HOB and 9-HDA, acting synergistically, show the aforementioned inhibitory effect [46]. Research conducted by Ronai et al. [47] revealed that the QMP has the ability to trigger programmed cell death in workers’ ovaries during the mid-oogenesis checkpoint. This occurrence results in the abortion of workers’ oocytes. However, these studies require further analysis due to the presence of high caspase activities in workers that were not exposed to the queen’s presence [47].

#### 3.1.5. Copulatory Behavior

The drones’ task in a colony is to maintain a constant temperature in the hive, ventilation and, above all, copulation. The QMP is described as a sexual pheromone that affects males in populations. Its task is to attract them to virgin queens and induce copulatory behavior in the Drone Congregation Area (DCA). Due to the fact that we classify this effect as one caused solely by smell, we can conclude that its reception is related to the anatomical differences between males and workers. In fact, drones show sexual dimorphism in the structure of their olfactory organs. In comparison to workers, males have a larger number of the pore plate sensilla. According to Jarriault and Mercer [48], this is a difference of about 16,000 sensilla Placodea (18,600 for drones and 2600 for workers). In addition, structures called macroglomeruli can be seen in the structure of their olfactory lobe. They do not occur in workers. The main component of the QMP that induces copulatory behavior in drones is 9-ODA. Their antennae are more sensitive to this component than the antennae of workers. This fact is connected with the occurrence of macroglomeruli and the number of pore sensilla in drones. Each glomerulus is connected to a concrete OR sensillum for the genus. Due to their role, drones are not required to detect a large number of odors. The most developed glomerulae that receive information from sensilla Placodea are those that recognize only sex pheromones. They are therefore enlarged in relation to the others [41,48]. Brockmann et al.’s [49] experiment suggested that, in addition to 9-ODA, HDA enantiomers (9-HDA and 10-HDA) act as supporting components of the sexual attraction blend [49].

#### 3.1.6. Recognizing Nestmates

In a honeybee colony, workers collect food from the 21st day of their life. As a result, they fly outside the colony. Upon their return, they must be recognized by their sister workers. In the course of evolution, social insects, which include the honeybee, have developed a number of signals, the most important of which are chemical signals. They constitute the cuticular hydrocarbon profile [50,51]. Hydrocarbons present on the surface of the cuticle are mainly responsible for preventing the insect’s body from drying out. Their other role is to create profiles specific to a given colony. The composition and saturation of individual compounds included in the profile constitute a chemical recognition signal for members of one family [52,53]. The most important components of the cuticular hydrocarbon profile in terms of nestmate recognition are alkenes. These hydrocarbons were determined to be learnt and discriminated better than alkanes [54].

The cuticular hydrocarbon profile is influenced by various factors. These include genetics, environmental factors, diet and the presence of the queen’s mandibular pheromone [55]. Fan et al. [56] showed that forager bees treated with the QMP had a more similar profile to resident bees than untreated bees. This suggests that the QMP does not directly influence aggressive behavior toward strangers approaching the colony [56]. Vernier et al. [57] has determined that CHC-based recognition develops via colony-specific factors modulating the genetic similarity of its members [57]. However, the initial mechanism of nestmate recognition within large colonies remains unsolved.

### 3.2. Tergal Gland Pheromones

Tergal glands are also called pocket glands or Renner and Baumann glands. They are found in the colony only in the queen bee. In workers, they are almost completely reduced. The pheromone produced and secreted by these glands supports the effects of the QMP, which makes it crucial for the functioning of the colony.

#### 3.2.1. Location and Structure of the Gland

**Figure 6:**
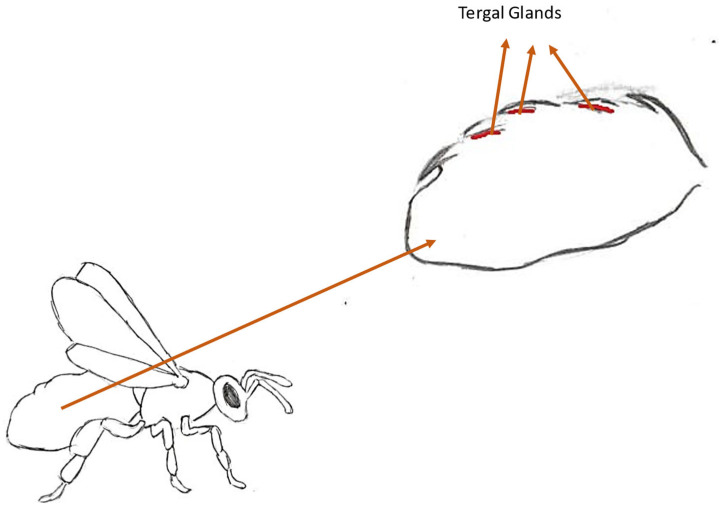
Location of the tergal glands.

The tergal glands are largely reduced in workers, to three to eleven cells, depending on the subspecies; for example, for *A. m. capensis*, the number of cells ranges from six to eleven, while for *A. cerana*, it ranges from one to three cells. In the mother, by comparison, the lobe of glandular cells is 2500 to 4500 µm long [10]. They are located under the membranes that connect tergites II to VI or, according to other sources, to V (Figure 6) [58,59,60]. Their structure is similar to the Nasonov gland. The excretory ducts are thin and long. They excrete secretion with pulsating movements. Each of the secretory cells has its own excretory duct. A mixture of substances spreads from the thickened funnel of the duct onto the surface of the intersegmental membrane. From there, it evaporates, creating a cloud of scent around the queen. It was found that there are no secretory tissues under tergite VI. Therefore, the substance most likely flows to this place from the other tergites. Despite being located under different tergites, the cells do not differ significantly in structure in queens. The only differences result from the size of the cell nucleus. The largest nuclei are found in the tergal glands located on tergite III, with the smallest on V [10,59]. Worker bees exhibit two types of tergal glands. Type A is located along tergites II-V on the anterior edge, with type B on the posterior edge of these tergites. Type B glands are bicellular and occur in both workers and queens, while type A glands consist of single cells. *A. m. capensis* possesses larger A-type cells in comparison to *A. m. scutellata*. Type A glands cells are directly related to the honeybee’s fat body and oenocytes (homologs of the mammalian liver) [58,61].

#### 3.2.2. Chemical Composition

The blend produced in tergal glands includes long-chain esters, unsaturated hydrocarbons and saturated and long-chain fatty acids. The mixture contains three esters that have primer and releaser effects. These are ethyl palmitate, ethyl oleate and ethyl stearate [10,62]. In addition, the pheromone contains compounds such as palmitooleic acid, vaccenic acid, methylstearic acid, octadecanoic acid, lauric acid, alkanes and alkenes [60]. There is a difference in primer compounds between virgin and older queens. In queens, around the third to tenth day, decyl decanoate and long-chain decanoic acid esters occur, while older mothers’ blends contain (Z)-9-octadecenoic acid [20]. A subsequent chapter provides a description of the elaboration in that matter.

#### 3.2.3. Support Pheromone

In the case of the QMP, one of its functions is to induce a behavioral response of workers towards the queen, which has been called a behavioral set. Bees gather around the queen and groom her by licking the pheromone from her body. The entourage response is considered to be one of the short-term effects of this mixture. Tergal gland pheromones have been identified as aids in the release of scent-related effects [60] and neural pathways (primer effects) [10]. Their action affects the strengthening of cluster formation around the queen and also causes the attraction of drones during wedding flights [10,63]. Princen et al. [64] conducted an experiment showing the crucial role of the tergal gland pheromone in ovary development inhibition. Their experiment showed that tergal gland esters reduced the activation of workers’ ovaries sevenfold (from 32% in unexposed workers to 6.2% in exposed workers). However, the synergic effect of this blend with the QMP was present only for inducing retinue behavior. Full QMP blends showed no enhanced effect in reducing ovary activation compared to individual components or tergal esters [64].

#### 3.2.4. Dependence of Activity on Age

The attractiveness of the queen to the workers changes throughout her life. This is determined by changes in the activity of the tergal glands in connection with the specific stages of the queen’s life. One-day-old queens show less attractiveness to the workers than five-day-old queens. Differences can also be seen when comparing the size of their tergal glands. Five-day-old queens have larger and more active glands. As a result, the workers show more interest in the abdomen of these queens [65]. Seven-day-old queens embark on nuptial flights, while six-day-old queens experience a peak in secretory activity. As mentioned above, the secretion of this gland supports the action of the QMP, interacting synergistically with it. This fact explains the connection between the age at which the nuptial flight takes place and the peak activity of Renner’s glands on the eve of this event. After the nuptial flight, on the 18th day of the queen’s life, there is another moment of maximum secretion of the substance [10]. Queens usually lay eggs 5–15 days after hatching. However, this is not a continuous process. Between the first and second egg laying, worker bees form an entourage around the mother, feeding and cleaning her [66]. This may explain the second secretory peak of pheromones from the tergite glands. Azevedo et al. (2007) suggested that a queen exhibiting maximum secretory activity of Renner’s glands will be better accepted in a new colony that is in a stable state [65].

#### 3.2.5. An Exception to the Rule—The Rebels

While in a colony where the queen is present, the QMP inhibits the development of ovaries in workers, in queen-less colonies, there is a possibility of developing rebel bees. These bees are not focused on rearing larvae and working, but on reproduction. They resemble the queen in terms of some anatomical features. Standard workers have almost completely reduced tergal glands. The remaining cells of these reduced glands are much more fragile. They also tend to burst quickly. In rebel workers, the lobes of these cells are from 1500 to 3000 µm. However, compared to the number of cells in 200 µm^2^, there are almost twice as many of them as in queens (approx. 15–25 cells). The diameters of the nuclei of these glands are also smaller compared to the queen [10]. In the chemical composition of the secreted mixture, esters such as ethyl palmitate and ethyl oleate were found. These compounds occur in the Renner gland mixture from queens and induce retinue behavior. Therefore, the presence of these esters in reproductively oriented bees is reasonable [60].

### 3.3. Dufour’s Gland Pheromone

One theory on the origin of Dufour’s gland is that it evolved over 200 million years. It is a gland common to all Apocrita and Symphyta. It was first described by Dufour in 1841. The pheromone produced in this organ occurs in both worker bees and queens. It has different functions between the two castes [67,68]. The role of this compound in the queen is controversial and, despite years of research, is still unclear.

#### 3.3.1. Location and Structure of the Gland

Dufour’s gland is an abdominal gland. It is located in the proximal part (i.e., facing the limb attachment) of the abdomen. It is an ectodermal gland. In bees, unlike ants, the excretory canal does not open into the stinger sheath, but into the dorsal wall of the vagina [68,69,70]. The morphology of this gland itself may vary between species or even genera. In all of them, however, it is described as a tube made of muscle and epithelium. Due to its structure, it is classified as a class I gland (developed through evolution from epidermal cells that have gained secretory abilities). It also has connections with structures such as tracheae and nerves. The structure of the cells confirms its secretory functions. They contain significant amounts of reticulum, mitochondria and secretory vesicles [68]. Queens’ Dufour glands produce larger volumes of secretion in comparison to those of workers (12 times larger) [70].

#### 3.3.2. Chemical Composition

Despite the differences in the chemical composition of this pheromone between the castes of the honeybee, it consists of only two basic compounds. These are specific hydrocarbons and esters. In workers, odd-numbered n-alkanes from C23 to C31 have been identified. The queen’s secretion contains additional substances in the form of long-chain esters. These include, among others, tetradecyl hexadecenoate, tetradecyl hexadecenoate, hexadecyl tetradecanoate, tetradecyl octadecanoate, octadecenoate and hexadecyl hexadecenoate. In the absence of a queen, when workers begin laying eggs, the composition of the pheromones produced by Dufour’s gland is altered. It then contains esters characteristic of queens [69]. The presence of hydrocarbons in the mixture is a feature considered characteristic of Hymenoptera [68].

#### 3.3.3. Queen’s Secretion

One of the first theses concerning the role of the gland for individuals and for the entire honeybee colony was the marking of eggs by the queen, due to small amounts of the queen’s esters being found on the egg surface. In the colony, workers are tasked not only with feeding and caring for the queen, but also with protecting her and her offspring. Due to the lower percentage of kinship between the offspring of workers compared to the offspring of the queen, workers protect her eggs. If workers appear in the hive and lay their own eggs, the remaining bees eliminate them. According to research conducted by Katzav-Goznansky [69], workers laying eggs in a colony without a queen have esters on their egg shells that imitate the queen’s esters. The possibility of marking with the secretion of Dufour’s gland remains an unresolved issue. In addition to the function of marking eggs, the queen’s secretion has been shown to have another function in the course of research. While we know that the QMP is a pheromone that induces the formation of a network of workers around the queen, Dufour’s gland has the same function. It has been shown that the secretions of the abdomen have a greater effect on workers than the secretions of the thoracic and head parts. Katzav-Goznansky et al. [69] conducted an experiment showing that the secretion of this gland not only induces networking behavior but at the same time is more attractive to workers than the secretion of the same gland from other workers. As a result of the research, a theory was put forward that the secretion from the queen’s Dufour gland serves to signal the queen’s reproductive potential. In the secretion of the same gland, workers who have undertaken reproduction during their lives have similar compounds to those produced by the queen. This is one of the arguments in support of the above thesis [69].

### 3.4. Nasonov’s Pheromone

Over the years, the secretion of the Nasonov gland has been variably classified from a pheromone to a general attractant. This gland was discovered in 1883 by the zoologist Nikolai Viktorovich Nasonov and was named after him. Today, this pheromone is considered an attractant and an indicator.

#### 3.4.1. Place of Synthesis and Method of Exposure

**Figure 7:**
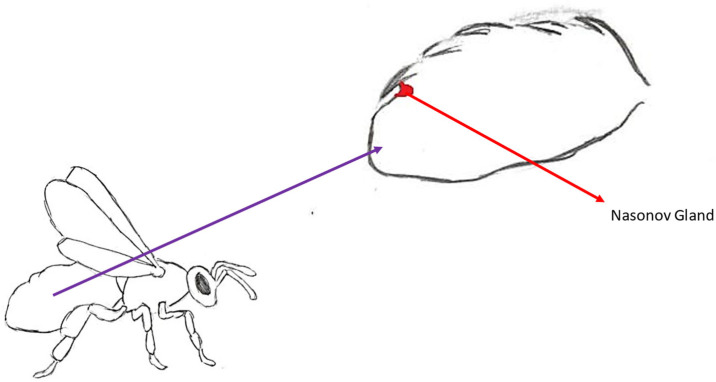
Location of the Nasonov gland.

Nasonov’s gland is located between tergites VI and VII in the upper part of the abdomen, between the intersegmental membrane (Figure 7). This gland is found only in workers [10]. It consists of cells that form a package in workers. Individual cells are large, and their nucleus is located centrally. They extend to a length of about 2000 µm [71]. Since the pheromone itself is highly volatile, its release is often aided by wing fanning. The channel is covered on the upper side by tergite VI. Lifting the abdomen allows the channel to be uncovered and the pheromone to be released into the air [71]. Newly emerged bees do not produce large amounts of this pheromone. This is most likely related to the division of functions in workers depending on their age. A 21-day-old worker is assigned to the exit to search for food, while the gland reaches its peak production at the age of 28 days [10].

#### 3.4.2. Chemical Composition

The mixture defined as Nasonov’s pheromone consists of seven key compounds. They belong to the group of terpenoid compounds. We can distinguish geraniol, (E)-citral, (Z)-citral, geraniic acid, nerolic acid, (E,E)-farnesol and nerol. Despite the fact that the entire mixture is volatile, its individual components differ in the degree of this property. Geraniol and (E,E)-farnesol are not present in blend of newly emerged bees. The concertation of geraniol increases to 0.3 µg during the first 10 to 17 days [66]. In addition, a similar mixture of components is found in the scents of some flowering plants. This increases the probability of confirming the thesis about the attractant properties of this pheromone [72].

#### 3.4.3. Luring to a New Home

The secretion of the Nasonov gland is an attractant. Workers use it when searching for a nest for the swarm. When the first workers find a suitable place for a new nest, a scout returns to the original hive. After returning, they use the so-called bee dance to communicate the distance to the new place of residence. It should be noted that the dance itself does not indicate the exact location of the new place, but only the area in which it is located. For this reason, the remaining group of scouts remains in place and spreads the Nasonov pheromone, signaling the exact place so that newly arrived workers can make the decision to move [73].

#### 3.4.4. Back to the Hive

Foraging bees flying out for nectar may have problems with orientation when returning to their hive. There may be many reasons for this disorientation, from illness to people removing landmarks encoded in the bee’s memory. It should also be remembered that, depending on the flight altitude, the bee may incorrectly determine the distance it traveled. Therefore, workers secreting Nasonov’s pheromone work in front of the hive/nest. In this place, it most likely mixes with odors from the hive, showing the foraging bees the way back [10,73]. This signal is also a clue for the queen, who returns to the hive after the nuptial flight [74].

#### 3.4.5. Nasonov for the Rebels

Due to the fact that the described gland occurs only in worker bees and is associated with their work for the hive, its structure in rebel bees is different than in workers with inhibited development of ovaries [44]. Granular cell packets in rebel bees stretch to a shorter length (about 800–1000 µm). In addition, the diameter of the cell nuclei in these individuals is smaller than in workers in which ovarian development was inhibited [72].

### 3.5. Alarm Pheromone

Apart from the QMP, the alarm pheromone is one of the best-known bee pheromones. It consists of over 40 components and when released, it emits a characteristic banana-like scent. Apart from causing aggression as a behavioral response (nipping, nudging, pulling hairs on the victim’s body and, finally, stinging), it is also responsible for an increased respiratory rate in the bee [75]. Its other name is the SAP (sting alarm pheromone). It works in a similar way to the MAP (mandibular alarm pheromone). However, it is used in the case of “larger threats”, and its synthesis takes place in the gland connected to the sting elements. Koschevnikov’s gland (which is the pheromone-producing gland) is present in both queens and workers; however, its blend composition and function differs for both castes. While workers produce this pheromone to alarm other workers about danger, the queen uses it as a supportive blend of the QMP for the first year of life [20].

#### 3.5.1. Place of Synthesis

**Figure 8:**
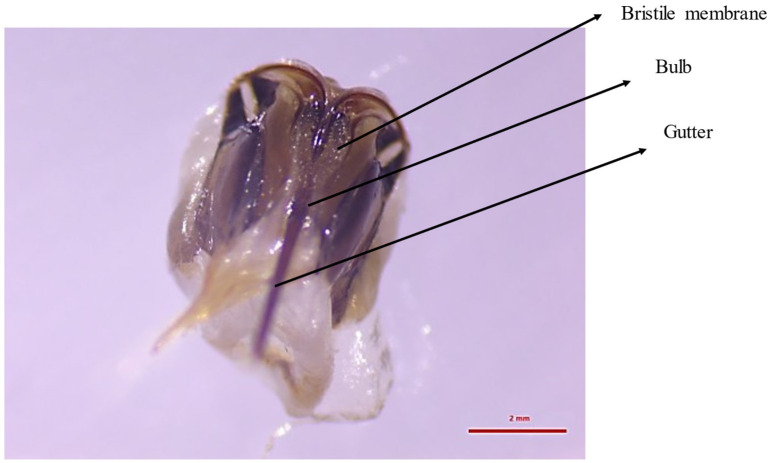
Sting morphology highlighting the bulb, gutter and membrane.

The sting consists of 10 elements. They include those significant for the SAP: a gutter that widens at the base, creating a bulb; a bristle membrane that envelops the bulb; and a pair of square plates, which develop from tergite IX (Figure 8) [10,76]. Each of these plates contains secretory cells of the Koschevnikov gland. On the lateral surface of this plate, it is visible as a granular mass [77]. These cells are surrounded by a chitin shell that contains numerous pores. An alarm pheromone is released from the interior of the cells into these pores. This is possible thanks to short tubes connected to the pores. The released secretion flows onto the bristle membrane located above the bulb. This membrane provides an increased surface for the dispersion of the pheromone, which evaporates from it. The irritating substance accumulates in the bulb during stinging [10].

#### 3.5.2. Chemical Composition

The pheromone mixture of the worker SAP contains about 40 chemical compounds. The most important of them is isoamyl acetate (IAA), which induces aggressive behavior, leading to the final sting [78]. The influence of interactions between bees on the behavioral response associated with the occurrence of IAA was studied. The experiment established that IAA causes an increased aggressive response in the case of a group attack than in the case of an individual attack. However, it cannot be ruled out that the presence of another bee acts additively rather than synergistically [79]. In addition to isoamyl acetate, the mixture also contains compounds such as n-butanol, 1-hexanol and butyl acetate [10]. It is the latter compound that gives the mixture a scent reminiscent of bananas. In the queen, 28 pheromone components were found that are not synthesized in workers. They consist of acids, alkanes, alkenes and alcohols. This fact allows us to conclude that this gland in the queen plays a different role than in workers. It has been determined to be consistent with the action of the QMP by attracting worker bees to the queen. The Koschevnikov gland disappears in queens after 1 year of age [20].

#### 3.5.3. Aggressive Behavior

**Figure 9:**
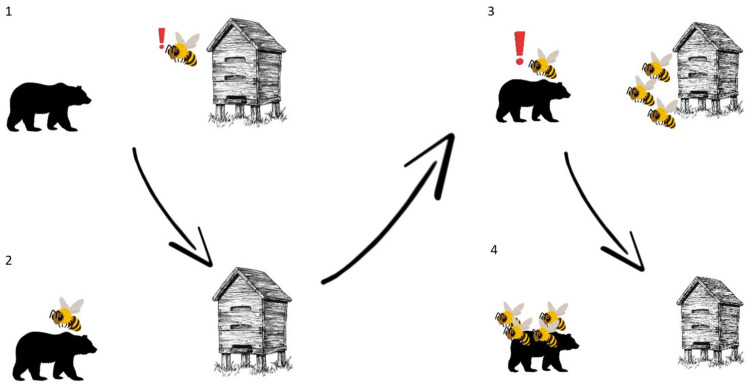
Scheme of an attack caused by SAP extraction. 1. A guard bee detects a potential threat near the hive. 2. A guard bee inspects and assesses the threat. 3. A guard bee release SAP. 4. Recruited bees collectively proceed with the attack.

The SAP is secreted by worker bees in the event of a threat identified as “larger” (an animal of a size that prevents one bee from eliminating it). The MAP is used to signal the need to defend against a smaller threat (such as other small insects). The aggressive reaction to alarm pheromones takes place in several stages (Figure 9). After a potential threat appears, the guard bee flies in to assess the level of threat and as a result secretes the MAP or SAP. If the threat is a larger predator, e.g., a human, the SAP is secreted. The remaining workers receive the odor stimulus and move towards the threat, starting the attack and stinging. Ramirez-Moreno [80] conducted an experiment that showed the importance of presence of the SAP during attacks against threats. While testing attacks in pairs of bees from different colonies, the highest increase constituted a rise from 0% to 83% in the occurrence of stinging. Furthermore, it was pointed out that guarding bees are more sensitive to the SAP during summer than winter. Therefore, the significance of the SAP is higher when more guarding bees are present at the nest entrance (when resources are not limited) [80]. Due to the structure of the sting, after the attack, it is anchored in the soft skin together with the last nerve ganglion, which leads to the release of a larger amount of alarm pheromone and a more massive attack [10,78].

### 3.6. Tarsal Gland Pheromone

The secretion of tarsal glands has been widely studied in the case of bumblebees and stingless bees (*Melipona*). The examination of these glands in honeybees is extremely outdated and barely mentioned in recent studies on the species. Glands producing this pheromone were first described by Arnhart and named after him, although his studies did not reveal the pheromonal properties of the secretion. Surprisingly, Arnhart glands are present in all castes, but the function of their secretion differs. The purpose of the secretion has been determined for both queens and workers, but it remains obscure in the case of drones.

#### 3.6.1. Secretion Site

**Figure 10:**
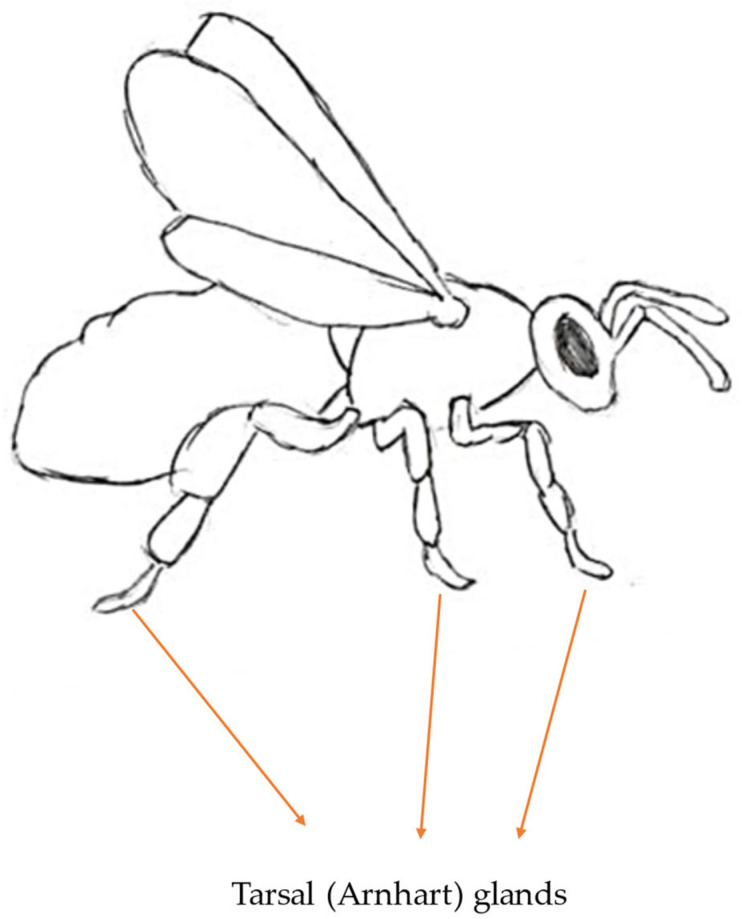
Location of the tarsal glands.

Legs of honeybees consist of the usual insect parts (coxa, trochanter, femur, tibia and tarsus). The last one (tarsus) can be divided into five segments, one of them being a set of three tarsomeres [10]. In the tarsus, Arnhart glands have been described. Each of the six legs possesses one tarsal gland in the shape of a flattened sac (Figure 10). One end is connected to the tendon of the pretarsal muscle. This distal end is described as blind in comparison to the proximal end, which opens on the ventral side of the junction, connecting the arolium (a soft, pad-like structure located between the claws of the tarsus) and the last tarsal segment. Each tarsal gland holds a cuticular sac (also named the glandular reservoir) within the cavity. The sac is connected to the pretarsal tendon, and it is created after the imaginal molt. The gland is formed by a unicellular layer of glandular epithelium created by palisade cells. The very apex of glandular cells is separated from the top layer of fibrillar endocuticular material by varicose subcuticular space. Glandular cells show a variety of organelle and nuclei distributions; for example, generally, regular-shaped nuclei are located in the cells in the lower third of glandular cells. Numerous cristae and microvilli line the vast crypts that connect to the subcuticular area on the glandular cells’ apical plasma membrane. The apical area of the glandular cells contains residual dense bodies, multivesicular bodies and pinocytotic vesicles. The Golgi apparatus is a separate organelle devoid of secretory granules, whereas the RER is fully established in the perinuclear and basal regions of glandular cells. Before the non-proteinaceous secretory product reaches the glandular pocket, it needs to cross several boundaries, such as the subcuticular space, the cuticular intima, the space between the intima and the cuticular wall and the cuticular wall. Interestingly, there were no exocytotic secretory structures found [10,81,82].

#### 3.6.2. Chemical Composition

While morphology of the tarsal gland does not differ between castes, its compound mixture does. Lenky et al. [83] established that the tarsal gland secretion in queens contains twelve specific compounds, with eleven workers and one in drones. Furthermore, there are differences in the rate of secretion between young queens, older mothers (with six-month old queens’ rates being higher) and workers (around 10–15 times lower). The mixture of compounds includes alkanes, alkenes, alcohols, organic acids, ethers, esters and aldehydes [83,84].

#### 3.6.3. Queen’s Regin

The queen’s pheromones are specifically tailored to maintain her reign in the colony. Tarsal gland secretions do not differ. Lenky et al. [85] conducted an experiment to test the theory that the tarsal gland pheromone inhibits the construction of queen cups. During the swarming season, the number of queen cups varies between hives with higher and lower free volumes (80,960 mL vs. 20,240 mL). In a colony with a higher free hive volume, the number of queen cups was approximately 51 times lower than in a colony with a lower free hive volume (1.5 queen cups in an 80,960 mL colony vs. 77 cups in a 20,240 mL colony). Observations of queen movement allowed us to determine that in higher-density hives, the queen cannot leave a pheromonal mark on the bottom edges of the comb (where queen cups are located typically). Furthermore, from the results of a bioassay, it was observed that only a mixture of tarsal gland secretions and mandibular gland pheromones was able to inhibit the formation of queen cups. When applied separately, the pheromones provoke no such reaction [85].

#### 3.6.4. Worker’s Mark

The Nasonov pheromone has been previously described as an attractant for worker bees that helps with both orientation while foraging and returning to the hive. The tarsal gland pheromone works in a similar way. However, while Nasonov’s pheromone works for longer distances, the tarsal gland pheromone induces a certain behavior within a closer area. Butler et al., after a series of experiments, postulated that tarsal gland pheromones left by crawling bees at the entrance to the hive work as an attractant for foragers [86].

Foraging workers have been reported to leave tarsal gland pheromones while collecting nectar. It is well known that a dish filled with syrup attracts far more foraging bees than an empty dish. It is in connection with a trail of pheromones left by workers on the full dish. However, another set of experiments showed that tarsal gland secretions left a different sort of mark for foragers. Presumably, pheromones left behind on visited flowers inform other foragers that these particular plants do not possess nectar anymore and that there is no reason to land on them in the first place [87,88,89].

## 4. Conclusions

Despite its small size, the honeybee has a highly complex anatomy and physiology. Its life and interactions between individuals are subject to continuous regulation, in which the key compounds are the described pheromones. The queen secretes the most important pheromone, which regulates the work of the entire colony. The division of workers’ tasks by age is the result of its action. Communication between workers depends on pheromone mixtures secreted by individuals. Drones are also recipients of queen pheromones. The variety of compounds that make up their composition and specific physiological/behavioral responses is enormous. The absence of any of them in the colony would lead to a breakdown in social organization, which would ultimately lead to the inability of the entire colony to survive. Despite many years of research, new questions about their action still arise, and answers to these questions could bring significant improvements in both beekeeping and bee protection in an ever-changing environment. The literature review described provides the following information and raises questions:

- Antennae are an organ specialized in receiving environmental signals, including pheromones.
- Sensilla occurring on the antennae flagellum show significant differences, resulting from the type of stimuli received.
- Stimuli received by some of the sensilla types remain unknown.
- Honeybees possess specialized brain structures that process pheromone information (antennal lobe; mushroom bodies).
- Even though extensive testing has reached the cellular level, the reason for pairs of mushroom bodies occurring is yet to be solved.
- Castes show differences in the occurrence of sensilla types, as well as in the morphology of special brain structures.
- The presence of the QMP in a colony is essential for its proper functioning.
- The specification of QMP components affecting the inhibition of ovarian development remains a topic to be explored.
- HDA enantiomers show a possible supporting effect for 9-ODA during sexual attraction of drones.
- The secretion of tergite glands is supportive of the QMP.
- Dufour gland function in the queen remains a contentious issue.
- The role previously assigned in egg marking has been called into question and replaced by a theoretical signaling of the queen’s reproductive capabilities.
- Nasonov’s pheromone enables the worker to orientate herself when leaving to forage and returning to the nest.
- The sting alarm pheromone enables more guard bees to be involved in nest protection.
- The tarsal gland pheromone has been thoroughly researched, although there is a lack of recent research focusing on its composition and properties.
- Its function for drones remains a mystery.

Further studies on pheromones will allow us not only to answer these questions but also to use existing knowledge in the conservation of honeybee species.

## Figures and Tables

**Table 1:** Comparison of information on all types of sensilla.

Type of Sensilla	Feature		
	Location on antennae	Shape	Type of stimulus
**Trichodea**	Every annuli of the flagellum.	Hair-like. Subtype A: thin and straight. Sybtype B: tapered and slightly bent. Subtype C: thin and arched. Subtype D: thick and strongly bent.	Mostly mechanoreceprors, with subtype A desribed as olfactory.
**Placodea**	3rd to 10th segments.	Oval pores in the cuticule, 9 × 6 µm thin.	Olfactoryreceptors.
**Basiconica**	4th to 10th flagelomers on the dorsal side and 8th, 9th and 10th on the ventral side; not present in drones.	Peg-like with nearly flat ends and numerous pores.	Gustatory and olfactory receptors.
**Coleoconica**	6th to 10th flagellomeres.	Externally grooved projection in a wide pit with a circular shape.	Hygroreceptor.
**Coleocapitular**	Tip of each antennae.	Located in a cavity in the cuticle. In the hole with a diameter of about 1.4 µm, there is a mushroom-shaped protrusion.	Hygro- and thermoreceptor.
**Campaniform**	Dorsal side of the 9th through 10th segments.	Disks with a flat and oval design. The cross-section resembles a bun with a dome.	Mechanoreceptors (register external skeleton stretch), presumably hygro- and thermoreceptors.
**Ampullacea**	6th to 10th flagellomeres, disregarding the ventral side. Often occur near Sensilla Coleoconica.	Holding a small peg with a sculpted surface inside a tiny aperture.	Presumably detect carbon dioxide levels.
**Chaetica**	3rd, 8th, and 10th flagellomeres for drones; presumably on the entire flagellum surface for workers.	Similar to Basiconica but with only one pore on the apex of the peg.	Gustatoryreceptors with the ability to detect aminoacids.

## Data Availability

Data are contained within the article.
